# Methanol Extract of *Clavularia inflata* Exerts Apoptosis and DNA Damage to Oral Cancer Cells

**DOI:** 10.3390/antiox11091777

**Published:** 2022-09-08

**Authors:** Yin-Yin Hsu, Ya-Ting Chuang, Ching-Yu Yen, Ming-Ya Cheng, Ching-Yeu Chen, Yuan-Bin Cheng, Hsueh-Wei Chang

**Affiliations:** 1 Graduate Institute of Medicine, College of Medicine, Kaohsiung Medical University, Kaohsiung 80708, Taiwan; 2 School of Dentistry, Taipei Medical University, Taipei 11031, Taiwan; 3 Department of Oral and Maxillofacial Surgery, Chi-Mei Medical Center, Tainan 71004, Taiwan; 4 Department of Marine Biotechnology and Resources, National Sun Yat-sen University, Kaohsiung 80424, Taiwan; 5 Department of Physical Therapy, Tzu-Hui Institute of Technology, Pingtung 92641, Taiwan; 6 Center for Cancer Research, Kaohsiung Medical University, Kaohsiung 80708, Taiwan; 7 Department of Medical Research, Kaohsiung Medical University Hospital, Kaohsiung 80708, Taiwan; 8 Department of Biomedical Science and Environmental Biology, College of Life Science, Kaohsiung Medical University, Kaohsiung 80708, Taiwan

**Keywords:** soft corals, marine natural product, oxidative stress, antiproliferation, oral cancer

## Abstract

Antiproliferation effects of *Clavularia*-derived natural products against cancer cells have been reported on, but most studies have focused on identifying bioactive compounds, lacking a detailed investigation of the molecular mechanism. Crude extracts generally exhibit multiple targeting potentials for anticancer effects, but they have rarely been assessed for methanol extracts of *Clavularia inflata* (MECI). This investigation aims to evaluate the antiproliferation of MECI and to examine several potential mechanisms between oral cancer and normal cells. A 24 h MTS assay demonstrated that MECI decreased cell viability in several oral cancer cell lines more than in normal cells. *N*-acetylcysteine (NAC), an oxidative stress inhibitor, recovered these antiproliferation effects. Higher oxidative stress was stimulated by MECI in oral cancer cells than in normal cells, as proven by examining reactive oxygen species and mitochondrial superoxide. This preferential induction of oxidative stress was partly explained by downregulating more cellular antioxidants, such as glutathione, in oral cancer cells than in normal cells. Consequently, the MECI-generated high oxidative stress in oral cancer cells was preferred to trigger more subG1 population, apoptosis expression (annexin V and caspase activation), and DNA damage, reverted by NAC. In conclusion, MECI is a potent marine natural product showing preferential antiproliferation against oral cancer cells.

## 1. Introduction

Oral cancers lead to high morbidity globally, especially in South Central Asia [1]. The occurrence for males is twice that of females for oral cancer [1]. Besides surgery and radiotherapy, chemotherapy provides an alternative or supportive way of curing oral cancer but frequently generates adverse effects [2]. Identifying a low side-effect anticancer drug is necessary.

Crude extracts containing diverse bioactive compounds may exhibit targeting to generate synergistic anticancer effects different than separate compounds [3,4]. For example, the methanol extract of *Usnea barbata* (MEUB) [5] shows at least 10-fold effectivity in oral cancer cells compared to its main bioactive compound (usnic acid) in melanoma cells [6]. Moreover, several natural product extracts provide selective antiproliferation against cancer cells while generating low side effects on normal cells. For example, aqueous extract of *Scutellaria baicalensis* [7], manoalide [8], and fucoidan [9], were reported to inhibit more cancer cell proliferation than normal cells.

Marine invertebrates are abundant resources containing diverse and unique bioactive compounds with anticancer effects [10]. Soft corals are marine invertebrates without internal skeletons and are rich in several bioactive compounds showing anticancer effects [11,12,13]. For example, several *Clavularia*-derived bioactive compounds showed antiproliferation effects against leukemia [14], liver [15], colon [16], prostate [17], and lung cancer cells [13]. However, the detailed mechanisms of action have not been investigated, and their bioactive compounds may have been extracted at a low yield.

Here, we focus on evaluating the anticancer function of the crude extract of the octocoral *Clavularia inflata* (*C. inflata*) collected from Green Island, located in southeast Taiwan. Therefore, the present work aims to inspect the modulating proliferation ability of the methanol extract of *C. inflata* (MECI) against oral cancer cells. The cell safety of MECI is also examined by testing its proliferation impacts on non-malignant oral cells. Detailed mechanisms of apoptosis induction and DNA damage were investigated for the first time.

## 2. Materials and Methods

### 2.1. MECI Preparation

Specimens of *C. inflata* were collected in May 2021 in Green Island, Taiwan, using SCUBA diving, and the voucher specimen (collection number CI2021) is given. The animal material was immediately frozen and transported to the Department of Marine Biotechnology and Resources, National Sun Yat-sen University, Kaohsiung, Taiwan. The coral sample was lyophilized and extracted by ethanol at room temperature three times. The EtOH extract was evaporated to provide an organic extract (124.6 kg). This extract was subjected to solvent partitioning between EtOAc and water. The EtOAc layer (34.8 g) was then dissolved in 75% aqueous MeOH and partitioned with hexane. The 75% aqueous MeOH layer of *C. inflata* was named MECI. MECI was dissolved in DMSO for further experiments. All experiments with or without MECI contained the same concentration of DMSO (0.1%).

### 2.2. Isolation of (1R*,12R*)-Dolabella-4(16),7,10-triene-3,13-dione (CI-A)

The 75% aqueous MeOH layer (11.1 g) was applied to flash chromatography on silica gel, stepwise eluting with a hexane−EtOAc-MeOH gradient system to afford nine fractions (CI-1 to CI-8). Fraction CI-2 (622.8 mg) was further isolated by silica gel open column stepwise eluted with hexane-acetone (40:1 to 0:1), and nine subfractions (CI-2-1 to CI-2-9) were obtained. Subfraction CI-2-6 (373.7 mg) was subjected to silica gel open column stepwise eluted with hexanes-EtOAc-MeOH (100:10:1 to 0:0:1), and the major diterpene (1R*,12R*)-dolabella-4(16),7,10-triene-3,13-dione (307.1 mg) was isolated. The Mass, ^1^H, and ^13^C NMR data were comparable to those recorded in the literature [16].

### 2.3. HPLC Analysis of MECI 

HPLC analysis was performed on a Shimadzu 40 series instrument equipped with an LC-40D quaternary pump, a DGU-405 degasser, a CTO-40S column oven, an SPD-M40A diode array detector, and a Phenomenex Luna C_18_ analytical column.

The chromatography processes were mentioned as follows: Solution A: H_2_O; solution B: MeCN; flow rate: 1.0 mL/min; 0 min: 40% solution B, 0–30 min: 40% to 75% solution B, 30–40 min: 75% to 80% solution B, 40–45 min: 80% to 100% solution B.

### 2.4. Cell Cultures, Viability, and Reagents

The oral cancer cell lines CAL 27 (tongue) (ATCC; Manassas, VA, USA), Ca9-22 (gingival), and HSC-3 (tongue) (JCRB Cell Bank; Osaka, Japan) were taken for experimentation. OC-2 (buccal mucosa) oral cancer cells [18] were supplied by Dr. Wan-Chi Tsai (Kaohsiung Medical University, Kaohsiung City, Taiwan). The non-malignant oral cells Smulow–Glickman (S–G), derived from gingival epithelial, were chosen to examine drug safety. As described, the medium was obtained from Gibco (Grand Island, NY, USA) [8]. Promega MTS reagent (Madison, WI, USA) was applied to measure cell viability after 24 h treatment and detected at 490 nm [9]. *N*-acetylcysteine (NAC) [19,20,21] (Sigma-Aldrich, St. Louis, MO, USA) at 10 mM for 1 h pretreatment was chosen as a reactive oxygen species (ROS) scavenger.

### 2.5. Cell Cycle

Cellular DNA within 75% ethanol-fixed cells was reacted with 7-aminoactinmycin D (7AAD) (Biotium, Hayward, CA, USA) [22,23]. After performing Guava easyCyte flow cytometry (Luminex, Austin, TX, USA), the cell cycle was determined according to DNA content by Flow Jo 10 software (Becton-Dickinson, Franklin Lakes, NJ, USA).

### 2.6. Annexin V/7AAD

Cells were mixed with annexin V (1:1000)/7AAD (1 μg/mL) for 1 h at 37 °C [24] (Strong Biotech, Taipei, Taiwan). After performing Guava easyCyte flow cytometry, the annexin V (+)/7AAD (+ or −) events were specified for the apoptotic cell population [25].

### 2.7. Caspase 3/8/9 (Cas 3/8/9)

Apoptosis is generally triggered by activating caspases. The extrinsic and intrinsic apoptotic caspases (Cas 8 and Cas 9), as well as the final caspase executer (Cas 3), were detected with the peptide-based detection kit (OncoImmunin kits, Gaithersburg, MD, USA) [9,26]. After activation of these caspases, the activated peptides, in turn, generate fluorescence for flow cytometry analysis. These caspase-specific substrates were diluted 1000-fold and incubated for 1 h. Finally, these cells were taken for flow cytometry.

### 2.8. Oxidative Stress

Oxidative stress is commonly detected by measuring ROS and mitochondrial superoxide (MitoSOX), which were probed with 2′,7′-dichlorodihydrofluorescein diacetate (H_2_DCFDA) (Sigma-Aldrich, St. Louis, MO, USA) [27,28] and MitoSOX™ Red [9] for flow cytometry. Moreover, glutathione (GSH) was determined with flow cytometry using 5-chloromethylfluorescein diacetate (CMF-DA) (Thermo Fisher Scientific, Carlsbad, CA, USA) staining as described before [9].

### 2.9. DNA Damage

DNA damage was detected with γH2AX [29] and 8-hydroxy-2-deoxyguanosine (8-OHdG) [9]. The γH2AX antibody [30] was obtained from Santa Cruz Biotechnology (Santa Cruz, CA, USA). Subsequently, Alexa Fluor^®^488-secondary antibody (Cell Signaling Technology) and 7AAD were added for double staining. Additionally, 8-OHdG was examined with the 8-OHdG-FITC antibody (Santa Cruz Biotechnology). Finally, the as-prepared cells were subjected to flow cytometry.

### 2.10. Statistical Analysis

JMP 14 software (SAS Institute Inc., Cary, NC, USA) was used to assess statistical analysis. Non-overlapping lowercase letters between different groups indicate significant results.

## 3. Results

### 3.1. HPLC Profile of MECI and Major Component

**Figure 1:**
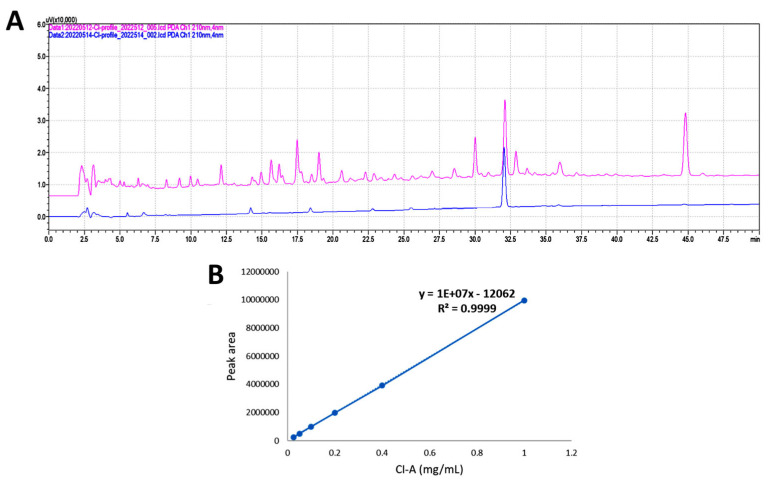
HPLC profile of MECI and major component. (**A**) The HPLC-UV (210 nm) fingerprint profile of MECI and (1R*,12R*)-dolabella-4(16),7,10-triene-3,13-dione are shown in red and blue, respectively. (**B**) The calibration curve of (1R*,12R*)-dolabella-4(16),7,10-triene-3,13-dione, namely CI-A (*n* = 3).

The HPLC-PDA fingerprint profile of MECI (red line) and major compound (1R*,12R*)-dolabella-4(16),7,10-triene-3,13-dione (blue line) is shown in Figure 1A. The retention time for (1R*,12R*)-dolabella-4(16),7,10-triene-3,13-dione is 32.046 min. The fingerprint profile of MECI also shows the peak of (1R*,12R*)-dolabella-4(16),7,10-triene-3,13-dione. The linear equation of major compounds was y = 10^7^ x − 12062 (R^2^ = 0.9999). The results showed that (1R*,12R*)-dolabella-4(16),7,10-triene-3,13-dione accounts for 3.17 % of MECI.

### 3.2. MECI Has Antiproliferation Effects on Oral Cancer Cells

**Figure 2:**
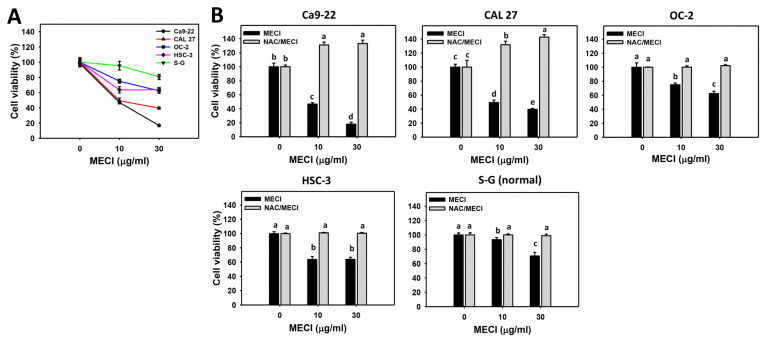
Antiproliferation. (**A**) Cell viabilities. Except for S-G cells, others were oral cancer cells. After 24 h MECI incubation, the cells were subjected to the MTS assay. (**B**) Impact of NAC on MECI-induced antiproliferation to oral cancer cells. After pretreating with NAC, cells were post-treated with MECI (0 (0.1% DMSO in the medium), 10, and 30 μg/mL) for 24 h. With or without MECI, all drug treatments were adjusted to contain the same DMSO (0.1%) in the medium. Data, mean ± SD (*n* = 3). For the same cell line, columns showing non-overlapping letters differ significantly (*p* < 0.05).

MECI downregulated the cell viability (%) for a panel of oral cancer cells (Figure 2A). For comparison, MECI-treated non-malignant cells (S–G) exhibited higher viability than oral cancer cells. The IC_50_ values of MECI for Ca9-22, CAL 27, HSC-3, OC-2, and S-G cells at 24 h MTS assays were 9.53 ± 0.18, 8.36 ± 0.86, 60.65 ± 3.08, 50.25 ± 4.08, and 75.15 ± 0.77 μg/mL, respectively.

Moreover, the impact of ROS on the controlling antiproliferation effects of MECI was assessed using an ROS inhibitor (NAC). As indicated, the antiproliferation effect of MECI in four oral cancer cell lines was inhibited by pretreating NAC (Figure 2B). Cells (Ca9-22 and CAL 27) highly susceptible to MECI were later used to assess the detailed antioral cancer mechanism.

### 3.3. MECI Has subG1-Incremental Effects

**Figure 3:**
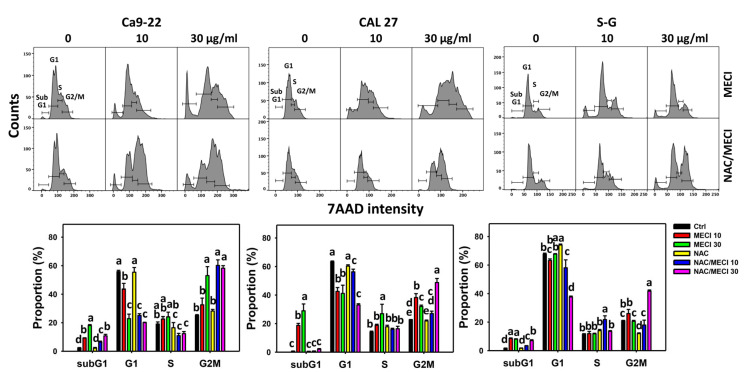
Cell cycle analysis. Except for S-G cells, others were oral cancer cells. After pretreating with NAC, cells were post-treated with MECI (0 (0.1% DMSO in the medium), 10, and 30 μg/mL) for 24 h and applied to the flow cytometry. Data, mean ± SD (*n* = 3). For the same cell line, columns showing non-overlapping letters differ significantly (*p* < 0.05).

Antiproliferation is partly affected by abnormal cell cycle progression [31,32] and subG1 is also an apoptosis-like indicator [33]. To assess the impact of MECI on cell cycle change, 7AAD-staining flow cytometry was performed (Figure 3). MECI increased the subG1% of oral cancer cells (Ca9-22 and CAL 27) (Figure 3), an indicator of fast screening for apoptosis. For comparison, non-malignant cells (S–G) exhibited lower subG1% than oral cancer cells. MECI decreased G1% and increased G2/M% in oral cancer cells but there was little change to S–G cells.

The impact of ROS on the regulation of the cell cycle progression by MECI was assessed using an ROS inhibitor (NAC). The subG1 accumulating effect of MECI in oral cancer and non-malignant cells was inhibited by pretreatment with NAC (Figure 3). Moreover, NAC increased the MECI-induced G2/M% compared to MECI alone at 30 μg/mL MECI treatment.

### 3.4. MECI Has Annexin V-Incremental Effects

**Figure 4:**
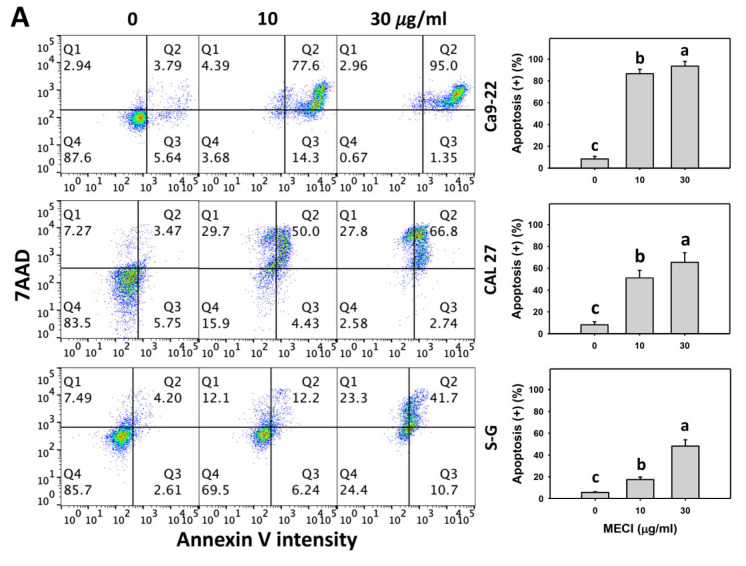

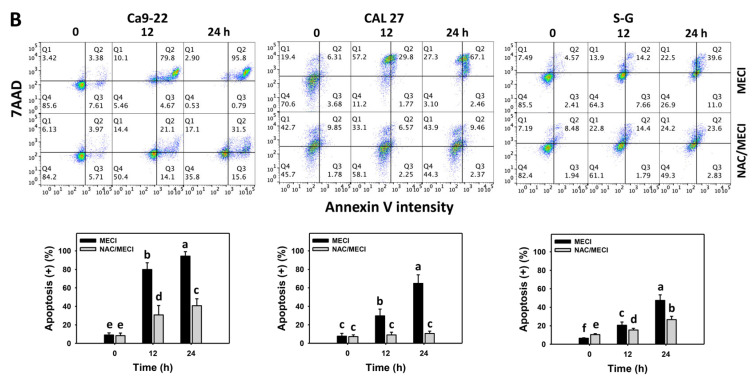
Annexin V-detected apoptosis. (**A**) Annexin V/7AAD. Except for S-G cells, others were oral cancer cells. After 24 h MECI incubation (0 (0.1% DMSO in the medium), 10, and 30 μg/mL), cells were subjected to flow cytometry. Annexin V (+)/7AAD (+/−) spots were specified for apoptosis (+). (**B**) Impact of NAC on MECI-induced apoptosis changes to oral cancer cells. After pretreating with NAC, cells were post-treated with MECI (30 μg/mL) for 0, 12, and 24 h. Data, mean ± SD (*n* = 3). For the same cell line, columns showing non-overlapping letters differ significantly (*p* < 0.05).

In addition to subG1-increasing effects, it is essential to assess apoptosis by additional methods. Using annexin V/7ADD detection, MECI enhanced the annexin V (+) % of oral cancer cells (Figure 4A), which is an advanced indicator of apoptosis. For comparison, non-malignant cells (S–G) showed lower annexin V (+) % than oral cancer cells.

The impact of ROS on the regulation of the annexin V level by MECI was assessed using an ROS inhibitor (NAC). The annexin V-increasing changes of MECI in oral cancer and non-malignant cells at time-lapse were inhibited by pretreatment with NAC (Figure 4B).

### 3.5. MECI Has Caspase-Activation Effects

**Figure 5:**
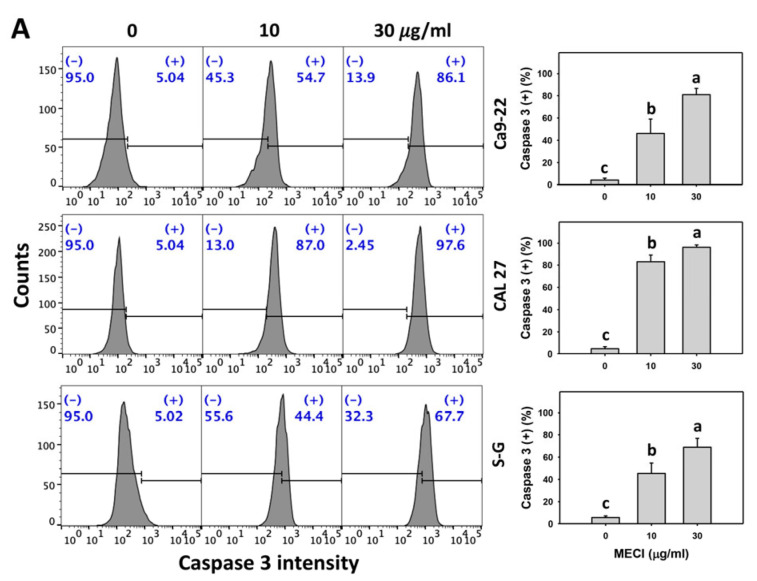

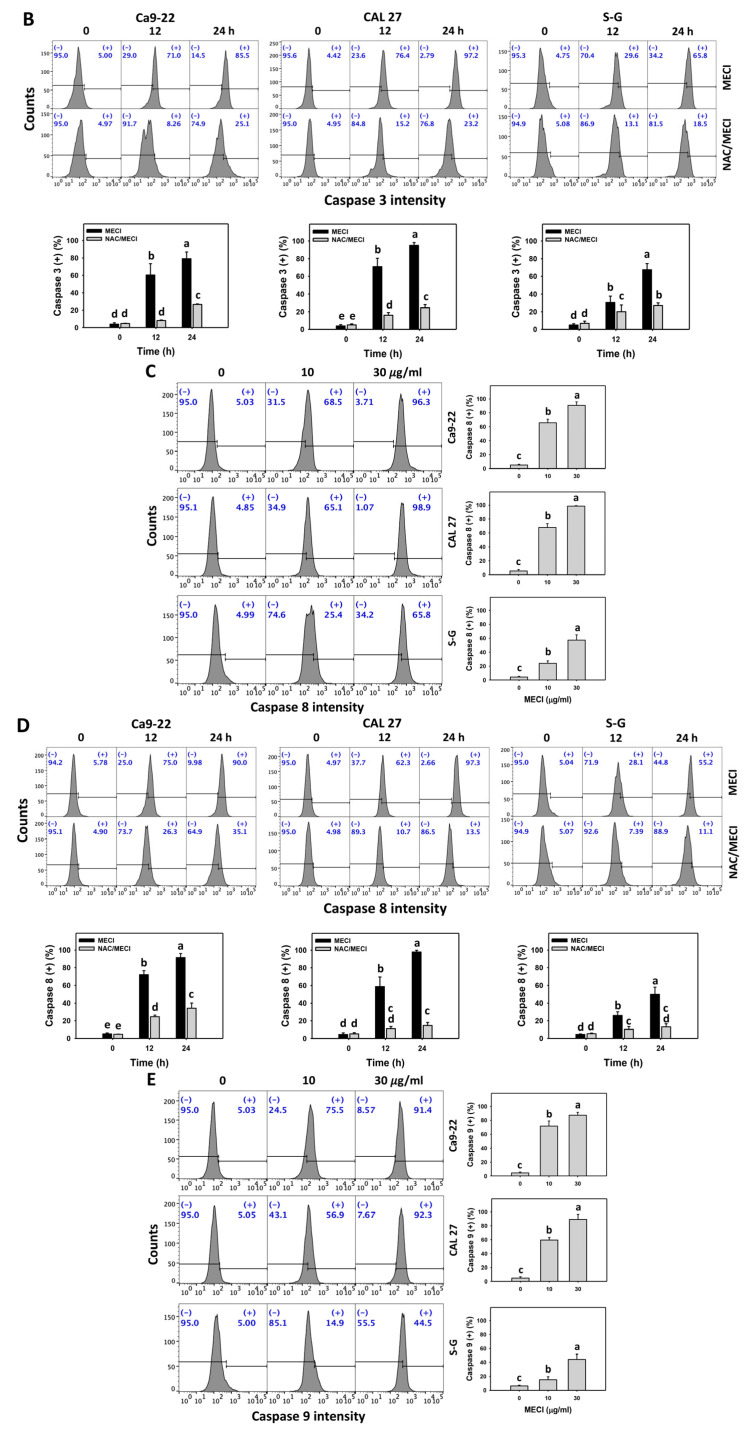

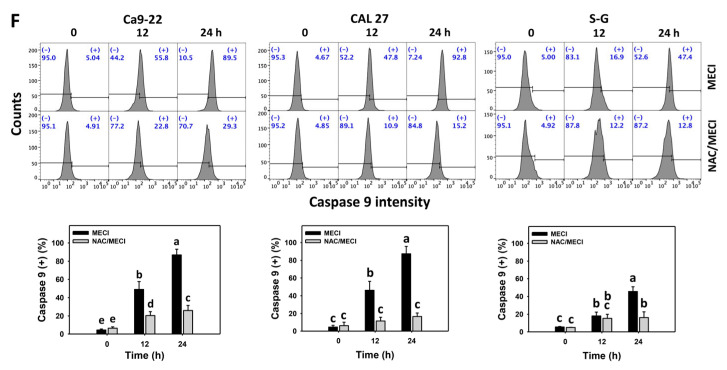
Cas 3/8/9 activation. (**A**,**C**,**E**) Cas 3/8/9 assay. Except for S-G cells, other cells were oral cancer cells. After 24 h MECI incubation (0 (0.1% DMSO in the medium), 10, and 30 μg/mL), cells were applied to Cas 3/8/9 assays. (+) spots were specified for Cas 3/8/9 (+). (**B**,**D**,**F**) Impact of NAC on MECI-induced Cas 3/8/9 change to oral cancer cells. After pretreating with NAC, cells were post-treated with MECI (30 μg/mL) for 0, 12, and 24 h. Data, mean ± SD (*n* = 3). For the same cell line, columns showing non-overlapping letters differ significantly (*p* < 0.05).

In addition to annexin V-increasing status, it is essential to assess apoptosis by more methods. Using caspase activation analysis (Figure 5A,C,E), MECI increased the Cas 3/8/9 (+) % of oral cancer cells, which is an advanced indicator of apoptosis in triggering caspase signaling. For comparison, non-malignant cells (S–G) exhibited lower Cas 3/8/9 (+) % than oral cancer cells.

The impact of ROS on the regulation of the Cas 3/8/9 (+) by MECI was assessed using an ROS inhibitor (NAC). The Cas 3/8/9 (+)-increasing status of MECI in oral cancer and non-malignant (S–G) cells at time-lapse was inhibited by pretreatment with NAC (Figure 5B,D,F).

### 3.6. MECI Has ROS-Incremental Effects

**Figure 6:**
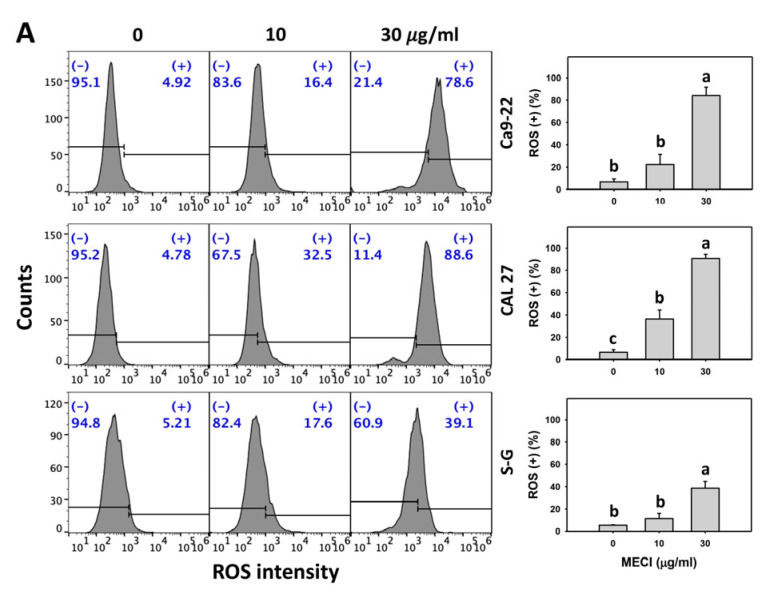

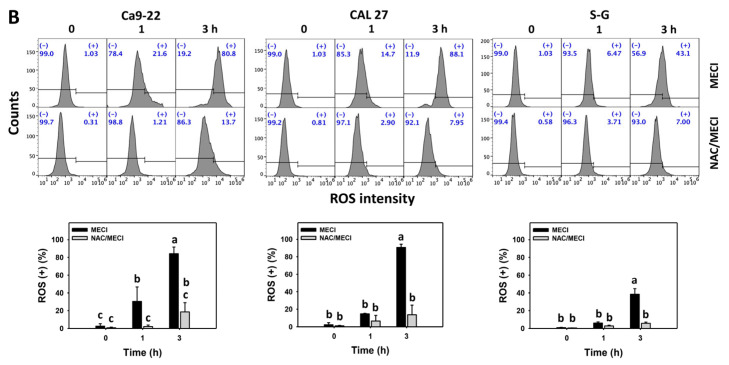
ROS assay. (**A**) ROS change. Except for S-G cells, other cells were oral cancer cells. After 3 h MECI incubation (0 (0.1% DMSO in the medium), 10, and 30 μg/mL), cells were applied to an ROS assay. (+) spots were specified for ROS (+). (**B**) Impact of NAC on MECI-induced ROS change to oral cancer cells. After pretreatment with NAC, cells were post-treated with MECI (30 μg/mL) for 0, 1, and 3 h. Data, mean ± SD (*n* = 3). For the same cell line, columns showing non-overlapping letters differ significantly (*p* < 0.05).

The effects of the antioxidant NAC were assessed above. Consequently, oxidative stress in terms of ROS was examined in MECI-treated cells. Based on graphs and statistics presented in Figure 6, there is a difference between the Ca9-22 (84.2%) and CAL 27 (90.7%) vs. S–G (38.6%) cells with regard to ROS generation after 30 μg/mL MECI treatment (Figure 6A). Using flow cytometry analysis, MECI increased the ROS (+) % of oral cancer cells (Figure 6A), which is an indicator of oxidative stress. For comparison, non-malignant cells (S–G) exhibited lower ROS (+) % than oral cancer cells.

The impact of ROS on the regulation of ROS levels by MECI was assessed using an ROS inhibitor (NAC). There is a difference between the Ca9-22 and CAL 27 vs. S–G cells with regard to the effect of NAC on MECI-generated ROS levels at 3 h MECI treatment (Figure 6B). The ROS-increasing status of MECI in oral cancer and non-malignant cells at time-lapse was inhibited by pretreatment with NAC (Figure 6B).

### 3.7. MECI Has MitoSOX-Incremental Effects

**Figure 7:**
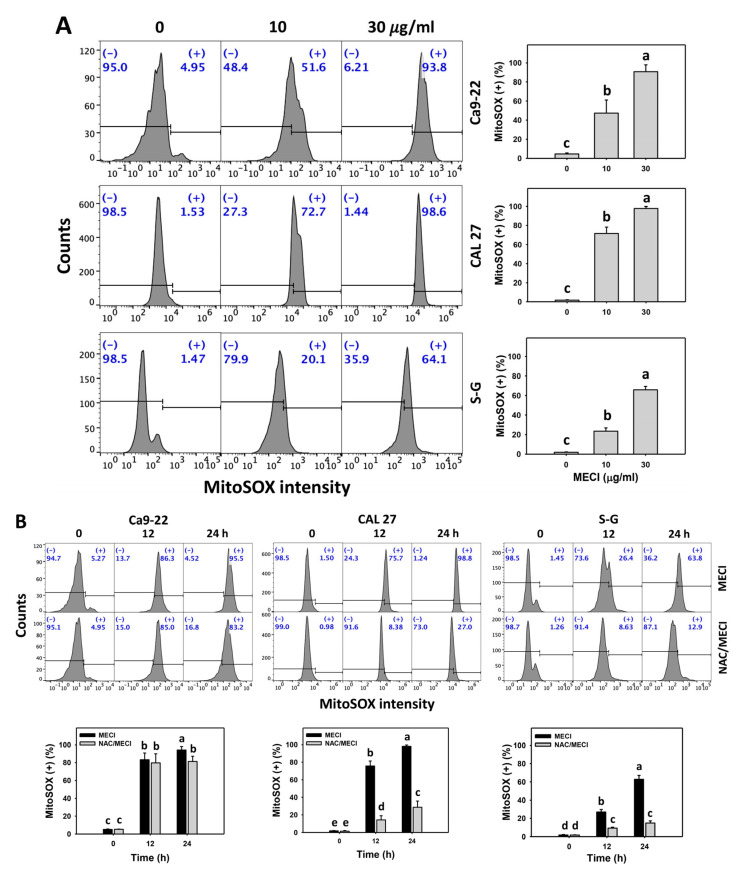
MitoSOX assay. (**A**) MitoSOX change. Except for S-G cells, other cells were oral cancer cells. After 24 h MECI incubation (0 (0.1% DMSO in the medium), 10, and 30 μg/mL), cells were subjected to a MitoSOX assay. (+) spots were specified for MitoSOX (+). (**B**) Impact of NAC on MECI-induced MitoSOX change to oral cancer cells. After pretreatment with NAC, cells were post-treated with MECI (30 μg/mL) for 0, 12, and 24 h. Data, mean ± SD (*n* = 3). For the same cell line, columns showing non-overlapping letters differ significantly (*p* < 0.05).

The oxidative stress, such as MitoSOX, was evaluated in MECI-treated cells. Based on graphs and statistics presented in Figure 7, there is a difference among the cell lines examined with regard to MitoSOX positivity after MECI treatment (Figure 7A). Using flow cytometry analysis, MECI increased the MitoSOX (+) % of oral cancer cells (Figure 7A), which is an indicator of oxidative stress. For comparison, non-malignant cells (S–G) exhibited lower MitoSOX (+) % than oral cancer cells.

The impact of ROS on the regulation of the MitoSOX levels by MECI was assessed using an ROS inhibitor (NAC). NAC eliminated MECI-induced MitoSOX positivity in the CAL 27 oral cancer and S–G normal oral cells more efficiently than in the Ca9-22 oral cancer cells (Figure 7B). The MitoSOX-increasing status of MECI in oral cancer cells at time-lapse was inhibited by pretreatment with NAC (Figure 7B).

### 3.8. MECI Has GSH-Decremental Effects

**Figure 8:**
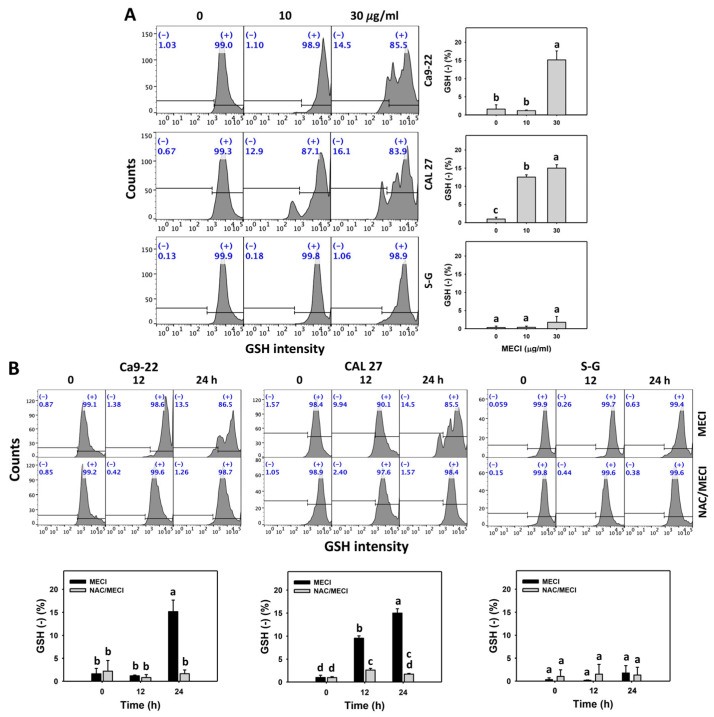
GSH assay. (**A**) GSH change. Except for S-G cells, other cells were oral cancer cells. After 24 h MECI incubation (0 (0.1% DMSO in the medium), 10, and 30 μg/mL), cells were applied to a GSH assay. (−) spots were specified for GSH (−). (**B**) Impact of NAC on MECI-induced GSH change to oral cancer cells. After pretreating with NAC, cells were post-treated with MECI (30 μg/mL) for 0, 12, and 24 h. Data, mean ± SD (*n* = 3). For the same cell line, columns showing non-overlapping letters differ significantly (*p* < 0.05).

The source of MECI-promoting oxidative stress was examined regarding the cellular antioxidant GSH changes in oral cancer cells. Based on graphs and statistics presented in Figure 8, there is a difference between the Ca9-22 and CAL 27 vs. S–G cells with regard to GSH (−) % after 30 μg/mL MECI treatment (Figure 8A). Using flow cytometry analysis, MECI increased the GSH (−) % of oral cancer cells (Figure 8A). For comparison, non-malignant cells (S–G) exhibited lower GSH (−) % than oral cancer cells.

The impact of ROS on the regulation of the GSH levels by MECI was assessed using an ROS inhibitor (NAC). NAC eliminated MECI-induced GSH negativity in the Ca9-22 and CAL 27 oral cells (Figure 8B). The GSH-decreasing status of MECI in oral cancer cells at time-lapse was inhibited by pretreatment with NAC (Figure 8B).

### 3.9. MECI Has DNA Damage-Incremental Effects

**Figure 9:**
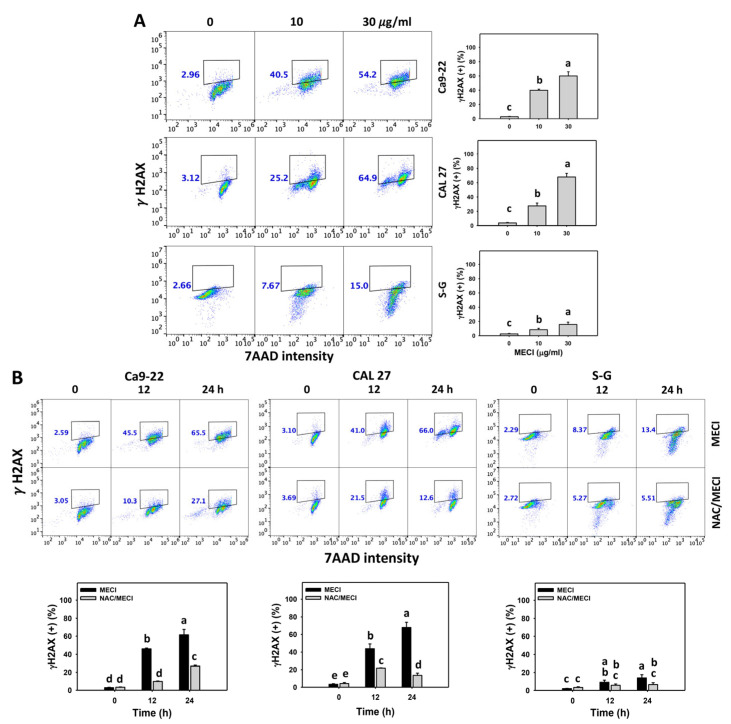
γH2AX assay. (**A**) γH2AX change. Except for S-G cells, other cells were oral cancer cells. After 24 h MECI incubation (0 (0.1% DMSO in the medium), 10, and 30 μg/mL), cells were applied to γH2AX assays. (+) spots were specified for γH2AX (+). (**B**) Impact of NAC on MECI-induced γH2AX change to oral cancer cells. After pretreatment with NAC, cells were post-treated with MECI (30 μg/mL) for 0, 12, and 24 h. Data, mean ± SD (*n* = 3). For the same cell line, columns showing non-overlapping letters differ significantly (*p* < 0.05).

**Figure 10:**
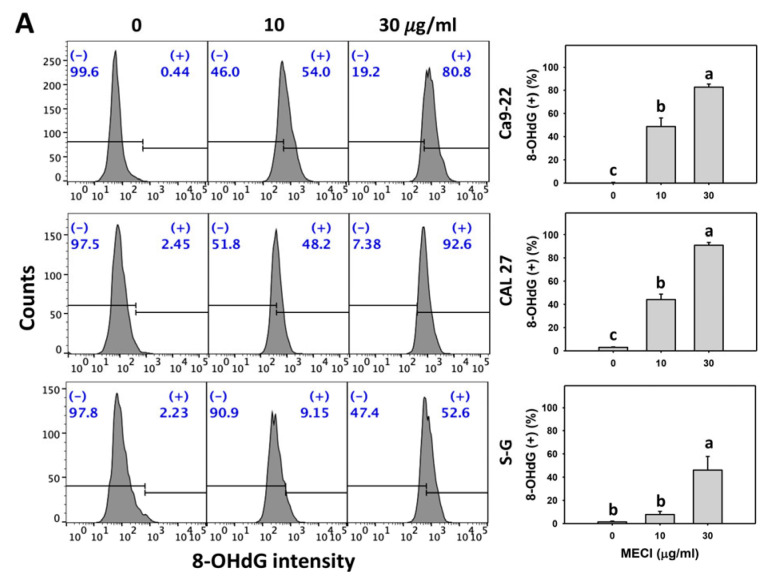

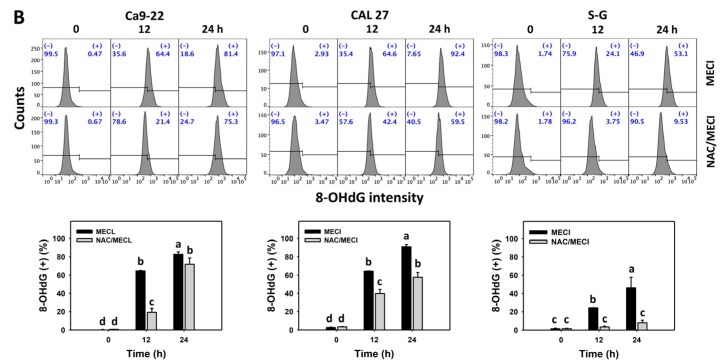
8-OHdG assay. (**A**) 8-OHdG change. Except for S-G cells, other cells were oral cancer cells. After 24 h MECI incubation (0 (0.1% DMSO in the medium), 10, and 30 μg/mL), cells were applied to 8-OHdG assays. (+) spots were specified for 8-OHdG (+). (**B**) Impact of NAC on MECI-induced 8-OHdG change to oral cancer cells. After pretreating with NAC, cells were post-treated with MECI (30 μg/mL) for 0, 12, and 24 h. Data, mean ± SD (*n* = 3). Columns showing non-overlapping letters differ significantly (*p* < 0.05).

Oxidative stress triggers DNA damage [34]. DNA damage such as γH2AX and 8-OHdG was examined in MECI-treated cells. Based on graphs and statistics presented in Figure 9 and Figure 10, there are differences among the cell lines examined with regard to γH2AX and 8-OHdG positivity after MECI treatment (Figure 9A and Figure 10A). Using flow cytometry analysis, MECI increased the γH2AX and 8-OHdG (+) % of oral cancer cells (Figure 9A and Figure 10A). For comparison, non-malignant cells (S–G) exhibited lower γH2AX and 8-OHdG (+) % than oral cancer cells.

The impact of ROS on the regulation of the γH2AX and 8-OHdG (+) levels by MECI was assessed using an ROS inhibitor (NAC). The γH2AX and 8-OHdG (+)-increasing status of MECI in oral cancer cells at time-lapse was inhibited by pretreatment with NAC (Figure 9B and Figure 10B). Based on graphs and statistics presented in Figure 9, the Ca9-22 and CAL 27 oral cells displayed the highest level of NAC-mediated protection against γH2AX formation by MECI. In Figure 10, the S–G normal oral cells displayed the highest level of NAC-mediated protection against 8-OHdG formation by MECI.

## 4. Discussion

The anticancer effects of MECI have rarely been reported. In assessing oral cancer cells, the detailed mechanisms of the antiproliferation ability of MECI were demonstrated for the first time. Moreover, this antiproliferation of MECI preferentially acts on oral cancer cells rather than non-malignant cells. The preferential induction of antiproliferation by MECI in oral cancer cells is attributed to several mechanisms, such as oxidative stress, cell cycle, apoptosis, and DNA damage. These mechanisms are discussed in the following.

### 4.1. MECI Suppresses Proliferation in Oral Cancer Cells

Several *Clavularia* extracts show antiproliferation ability concerning cancer cells. For example, ethyl acetate extracts from *C. viridis* and *C. australis* provided antiproliferation and apoptosis-inducible functions on oral cancer cells [35]. They showed IC_50_ ranging from 31.5 to 49.3 μg/mL at 18 h MTS assay for oral cancer SCC4, SCC9, and SCC25 cells. In the present study, the methanol extract of *C. inflata* (MECI)-treated oral cancer cells (Ca9-22 and CAL 27) showed IC_50_ values of 9.42 and 9.14 μg/mL at 24 h MTS assay. These findings suggest that MECI is more sensitive to oral cancer cells than *Clavularia* ethyl acetate extracts.

Crude extracts are a mixture of several bioactive compounds, showing cooperative targeting to induce synergistic antiproliferation effects compared to single compounds [3]. For example, the methanol extract of *Usnea barbata* (MEUS) showed IC_50_ values ranging from 1.4 to 6 μg/mL at 48 h MTS assay for oral cancer cells [5]. In comparison, usnic acid, the primary component of MEUB, showed IC_50_ values of 32.8 and 22.4 μg/mL at 48 h MTT assay for colon and lung cancer cells [36].

Similarly, some bioactive compounds derived from *Clavularia* also exhibit anticancer effects. *C. viridis*-derived 4-deacetoxyl-12-*O*-deacetylclavulone I showed an IC_50_ value of 2.5 μg/mL for prostate cancer cells (PC3) based on a sulforhodamine B assay at 48 h [17]. *C. viridis*-derived clavuperoxylides B showed an IC_50_ value of 5.23 μg/mL for lung cancer cells (A549) based on a CCK-8 assay at 72 h [13]. *C. viridis*-derived claviridic acid C showed an IC_50_ value of 7.78 μg/mL for gastric cancer cells (AGS) based on an MTT assay at 72 h [37]. Some *Clavularia*-derived compounds are more sensitive than the crude extract MECI, and some are similar.

Cisplatin, a common clinical anticancer drug, shows slightly higher sensitivity than MECI in oral cancer cells (Ca9-22) based on a 24 h MTS assay, e.g., IC_50_ values of 2.38 [38] and 9.42 µg/mL (Figure 2), respectively. However, the side-effect problem of cisplatin frequently occurs in clinical applications [39]. By contrast, MECI exhibits lower cytotoxicity in normal cells than in oral cancer cells (Figure 1). The character of preferential antiproliferation ability of MECI benefits the future application of oral cancer treatment.

### 4.2. MECI Generates Oxidative Stress in Oral Cancer Cells

Unbalancing redox homeostasis is a potent anticancer strategy [40,41]. Drugs with ROS modulating function may exhibit antiproliferation effects on cancer cells. Several natural products show antiproliferation of cancer cells associated with oxidative stress production [42]. Several literature reports support this rationale as follows. Isoaaptamine generates oxidative stress to inhibit breast cancer cell proliferation [27]. Notably, natural products such as manoalide [8] and fucoidan [9] can upregulate more oxidative stress generation and cause stronger antiproliferation against cancer cells than normal cells. Similarly, MECI-treated oral cancer cells show more ROS and MitoSOX levels than normal cells (Figure 6 and Figure 7).

In redox homeostasis, cellular antioxidants counteract the prooxidants [43]. Downregulation of cellular antioxidants may result in an overwhelming generation of oxidative stress [44]. For example, indoxyl sulfate causes GSH depletion in renal tubular cells to generate oxidative stress [45]. Fucoidan, the marine algae-derived polysaccharide, causes oxidative stress associated with downregulating GSH in oral cancer cells [9]. Moreover, MECI induces more GSH depletion in oral cancer cells than in normal cells (Figure 8). This GSH downregulation of oxidative stress is similar to that of MECI. This finding reveals that MECI showed preferential generation of oxidative stress against oral cancer cells.

In addition to cellular antioxidants, the enzymatic antioxidant signaling pathway also participates in maintaining redox homeostasis [43]. For example, nuclear factor erythroid 2-like 2 (NFE2L2), thioredoxin (TXN), catalase (CAT), superoxide dismutase 1 (SOD1), and heme oxygenase 1 (HMOX1) are common enzymatic antioxidant signaling proteins [46]. *NRF2*, *TXN*, and *HMOX1* genes were downregulated by fucoidan treatment in oral cancer, causing oxidative stress [9]. Pomegranate extract also downregulates *NFE2L2*, *TXN*, *CAT*, *SOD1*, and *HMOX1* gene expressions in oral cancer cells and induces oxidative stress [47]. Accordingly, it warrants a careful assessment of enzymatic antioxidant signaling in MECI-induced oxidative stress response in oral cancer cells.

### 4.3. MECI Generates Cell Cycle Redistribution, Apoptosis, and DNA Damage in Oral Cancer Cells

Overwhelming oxidative stress production is a promising treatment against cancer cells, accompanied by cell cycle arrest [48], apoptosis [49], caspase activation [50], and DNA damage [51]. Oxidative stress may trigger a DNA damage response. When DNA damage is too severe, cell cycle progression is prone to be arrested for cell cycle checkpoint examination. Once the DNA damage exceeds the tolerance, cells may process apoptosis [48].

Activation of checkpoint kinase 1 (CHK1) phosphorylates inactivates the phosphatase (cell division cycle 25; CDC25). In turn, it inactivates cell division cycle 2 (CDC2)-cyclin B1 complex, leading to G2/M arrest [52]. MECI declined the G1 phase population and caused G2/M arrest in oral cancer cells but not S–G cells. This warrants a detailed investigation of the response of the G2/M checkpoint in MECI-treated cells. Moreover, MECI caused differential cell cycle redistribution between Ca9-22, CAL 27, and S–G cells. It is possible that different doubling times of them may contribute to this difference in cell cycle changes. Additionally, different levels of subG1 population between Ca9-22 and CAL 27 cells were evidenced after MECI treatments. When cells become apoptosis, the chromosomal DNA molecules are cleaved by a caspase-dependent endonuclease such as caspase 3 [53], generating the sub-G1 population detected by flow cytometry. This caspase 3 activation appeared in the MECI-treated oral cancer cells (Ca9-22 and CAL 27) (Figure 5A,B).

Oxidative stress also triggers apoptosis [49]. For example, fucoidan exhibits preferential production of oxidative stress in oral cancer cells, leading to preferential apoptosis rather than in normal cells [9]. Similarly, MECI promotes higher subG1 accumulation (Figure 3) and causes more apoptosis (annexin V) in oral cancer cells than in normal cells (Figure 4). The molecular mechanism for improving apoptosis by MECI was explored with flow cytometry, demonstrating that MECI activates more extrinsic and intrinsic caspases (Cas 8 and Cas 9) and executor caspase (Cas 3) in oral cancer cells than in normal cells (Figure 5). Therefore, MECI provides a preferential induction of apoptosis in oral cancer cells.

Based on γH2AX and 8-OHdG [50], MECI-promoted DNA damage was further validated. MECI shows higher γH2AX and 8-OHdG levels in oral cancer than normal cells (Figure 9 and Figure 10), suggesting that MECI exhibits a preferential induction of DNA damage in oral cancer cells.

### 4.4. Function of Oxidative Stress in Antioral Cancer Mechanism of MECI

Although oxidative stress was upregulated, the crucial function of oxidative stress in drug treatment still needs to be validated by examining ROS removal using NAC pretreatment. Several literature reports prove that NAC recovers drug-induced oxidative stress-associated changes. For example, natural products such as fucoidan [9] and cryptocaryone [54] show oxidative stress-dependent mechanisms in oral and ovarian cancer cells, validated by NAC. In the present investigation, the MECI-promoting mechanism for oxidative stress, apoptosis, and DNA damage was blocked by NAC. Additionally, in the case of Ca9-22 and CAL 27 cell lines, NAC/MECI increases cell viability above 100% (Figure 1). It is possible that NAC (a GSH precursor) or GSH reacts with MECI, which improves the cell proliferation of oral cancer cells in addition to decreasing the MECI-induced oxidative stress. As described above, MECI exhibits preferential oxidative, apoptosis, and DNA damage changes, leading to preferential induction of antiproliferation for oral cancer cells. Consequently, MECI causes oxidative stress-dependent preferential antiproliferation mechanisms on oral cancer cells.

### 4.5. Limitation of MECI Study

Selective MECI-mediated killing of cancer cells compared to normal ones was demonstrated in the present study. However, the selective killing effects of MECI were not examined in other malignity types, limiting its application to other cancer cell treatments. This warrants an assessment of the antiproliferation effects of MECI on more malignant types in the future.

## 5. Conclusions

The present study identifies the anticancer effects of MECI for the first time, using the example of oral cancer cells. MECI demonstrates preferential induction of antiproliferation in oral cancer cells but less damage to normal cells. This preferential antiproliferation ability of MECI depends on oxidative stress, where MECI induces higher oxidative stress in oral cancer cells in examining ROS and MitoSOX than in normal cells. This character of MECI-induced preferential oxidative stress against oral cancer cells is accompanied by triggering more GSH depletion against oral cancer cells than in normal cells. Consequently, these MECI-treated oral cancer cells induce higher oxidative stress bursts and promote more apoptosis, caspase activation, and DNA damage than normal cells. These MECI-triggered oxidative-related mechanisms were suppressed by NAC pretreatment, suggesting that MECI-triggered oxidative stress-mediated antiproliferation, apoptosis, and DNA damage of oral cancer cells occurred.

## Data Availability

Data are contained within the article.
